# Effects of Gintonin-enriched fraction on the gene expression of six lysophosphatidic receptor subtypes

**DOI:** 10.1016/j.jgr.2021.02.006

**Published:** 2021-02-22

**Authors:** Rami Lee, Byung-Hwan Lee, Sun-Hye Choi, Yeon-Jin Cho, Han-Sung Cho, Hyoung-Chun Kim, Hyewhon Rhim, Ik-Hyun Cho, Man Hee Rhee, Seung-Yeol Nah

**Affiliations:** a Ginsentology Research Laboratory and Department of Physiology, College of Veterinary Medicine, Konkuk University, Seoul, Republic of Korea; b Neuropsychopharmacology and Toxicology program, College of Pharmacy, Kangwon National University, Chunchon, Republic of Korea; c Center for Neuroscience, Korea Institute of Science and Technology, Seoul, Republic of Korea; d Department of Convergence Medical Science, Department of Science in Korean Medicine, Graduate School, Kyung Hee University, Seoul, Republic of Korea; e Laboratory of Veterinary Physiology and Cell Signaling, College of Veterinary Medicine, Kyungpook National University, Daegu, Republic of Korea

**Keywords:** Differential LPA6 receptor subtype regulation, Ginseng, Gintonin, Mouse organs, Six LPA receptor subtypes

## Abstract

**Background:**

Gintonin, isolated from ginseng, acts as a ginseng-derived lysophosphatidic acid (LPA) receptor ligand and elicits the [Ca^2+^]_i_ transient through six LPA receptor subtypes (LPARSs). However, the long-term effects of gintonin-enriched fraction (GEF) on the gene expression of six LPARSs remain unknown. We examined changes in the gene expression of six LPA receptors in the mouse whole brain, heart, lungs, liver, kidneys, spleen, small intestine, colon, and testis after long-term oral GEF administration.

**Methods:**

C57BL/6 mice were divided into two groups: control vehicle and GEF (100 mg/kg, *p.o.*). After 21-day saline or GEF treatment, total RNA was extracted from nine mouse organs. Quantitative-real-time PCR (qRT-PCR) and western blot were performed to quantify changes in the gene and protein expression of the six LPARSs, respectively.

**Results:**

qRT-PCR analysis before GEF treatment revealed that the LPA6 RS was predominant in all organs except the small intestine. The LPA2 RS was most abundant in the small intestine. Long-term GEF administration differentially regulated the six LPARSs. Upon GEF treatment, the LPA6 RS significantly increased in the liver, small intestine, colon, and testis but decreased in the whole brain, heart, lungs, and kidneys. Western blot analysis of the LPA6 RS confirmed the differential effects of GEF on LPA6 receptor protein levels in the whole brain, liver, small intestine, and testis.

**Conclusion:**

The LPA6 receptor was predominantly expressed in all nine organs examined; long-term oral GEF administration differentially regulated LPA3, LPA4, and LPA6 receptors in the whole brain, heart, lungs, liver, kidneys, small intestine, and testis.

## Graphical abstract

**Figure:**
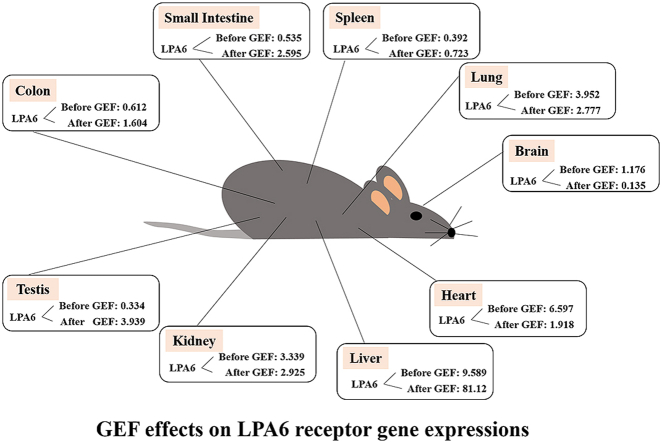

## 1 Introduction

Lysophosphatidic acid (LPA, 1-acyl-2-hydroxy-*sn*-glycero-3-phosphate) is a bioactive phospholipid present in plant and animal systems [1]. In plants, including medicinal herbs, LPA is an intermediate product for further phospholipid synthesis, such as phosphatidic acid and other glycophospholipids and phospholipids for the plasma membrane [2]. Some herbal medicines also contain some amount of LPA, depending on the species of herbal medicines [3]. In animal systems, LPAs act differently from that in plant systems because they act as lipid-derived growth factors [4]. LPA exists in body fluids and organs such as the brain, egg, plasma, saliva, stomach, intestines, and other tissues [2,5,6]. LPAs, which are derived from herbal medicines and animals, have diverse in vitro and in vivo biological effects in the neuronal and non-neuronal cells and organs. The primary cellular actions of LPAs in mammalian cells involve the regulation of the levels of cytosolic secondary messengers such as [Ca^2+^]_i_ or cAMP [7]. Thus, LPAs mediate Ca^2+^-dependent cell proliferation, differentiation, morphological changes, migration, and survival [7]. These effects are mediated by the activation of six different GTP-binding protein-coupled LPA receptor subtypes [7]. Although the six different LPA receptor subtypes are known, the role of the LPA1 receptor in the brain is relatively well characterized, and little is known about the distribution or roles of other LPA receptor subtypes in various organs except the brain and how these LPA receptor subtypes are regulated by exogenous LPA receptor ligand treatment.

Ginseng (*Panax ginseng* Meyer) has been used as a traditional herbal medicine for human well-being in Asian and Western countries [8]. In vitro and in vivo preclinical studies revealed that ginseng may attenuate brain aging, strengthen physical performance, and improve body stamina [7,9]. Ginseng has anti-inflammatory and antioxidant effects and improves metabolic diseases such as diabetes mellitus. The active ingredients of ginseng responsible for these diverse beneficial effects have also been reported, and they are mainly classified as saponin and non-saponin components [7,8].

Gintonin was recently isolated from the total saponin fraction of ginseng root, leaves, and stems [10,11]. Gintonin-enriched fraction (GEF) was later prepared via the ethanol extraction of ginseng through water fractionation, producing a higher yield than gintonin [7]. Gintonin is an active ligand at the LPA receptors because its primary active ingredients are LPAs, of which LPA C_18:2_ is abundant compared to other LPAs [12]. Gintonin contains large amounts of LPAs compared to other food and herbal medicines [2]. Thus, gintonin is a novel non-saponin and non-acidic polysaccharide isolated from ginseng [7]. Physiological and pharmacological studies showed that gintonin exhibits diverse functional effects in vitro through Ca^2+^- and cAMP-dependent signaling [7,13]. In vivo studies revealed that gintonin-mediated activation of the LPA–LPA receptor axis induces many beneficial brain functions through the facilitation of hippocampal synaptic transmission and brain-derived neurotrophic factor (BDNF) and vascular endothelial growth factor (VEGF) to prevent and attenuate neurodegenerative diseases [[14], [15], [16], [17], [18]].

However, little is known about the expression patterns of the six LPA receptor subtypes in mammalian organs and the long-term effects of GEF on the expression changes of the six LPA receptor subtypes in the organs. In the present study, we first examined the basal expression of the six LPA receptor subtypes in the whole brain, heart, lungs, liver, kidneys, spleen, small intestine, colon, and testis of mice and examined GEF-mediated regulation of the gene expression changes of the six LPA receptor subtypes in nine organs after long-term oral administration of GEF. We used quantitative real-time polymerase chain reaction (qRT-PCR) and western blot analysis. We further discussed the roles of GEF on the regulation of the gene expression of the six LPA receptors in mammalian organs.

## 2 Methods

### 2.1 Gintonin and molecular reagent preparation

GEFs were prepared as previously described [12]. LPA6 was purchased from OriGene Technologies, Inc. (Rockville, MD, USA). Anti-β-actin-HRP–conjugated antibody was obtained from Abcam (Cambridge, MA, USA), and goat anti-rabbit IgG–HRP-conjugated antibody (as a secondary antibody) was obtained from GeneTex (Irvine, CA, USA). For all molecular studies, the reagents, kits, and chemicals used were purchased from Bio-Rad (Hercules, CA, USA), TaKaRa (Tokyo, Japan), Intron (Seongnam, Republic of Korea), Invitrogen (Waltham, MA, USA), and Sigma-Aldrich (St. Louis, MO, USA).

### 2.2 Animals and experimental protocols

Four-week-old male C57BL/6 mice (18–20 g) were purchased from OrientBio (Chuncheon, Korea) for all experiments. The animals were maintained at a humidity level of 50 ± 5% under a 12-h/12-h light–dark cycle and were fed ad libitum. All experiments were performed in accordance with the Guidelines for the Care and Use of Laboratory Animals provided by Institute for Laboratory Animal Research (ILAR, 2010). This protocol was approved by the Institutional Animal Care and Use Committee of Konkuk University (No. KU17109). The mice were randomly divided into two groups (n = 6/group): control group and gintonin (GEF) at 100 mg/kg group (GEF100 group). The control group was treated with saline only, whereas the GEF100 group was administered GEF (dissolved in saline) at 100 mg/kg daily for 21 days. After drug administration, the mice were anesthetized with diethyl ether, and the organs were removed and frozen for further experiments.

### 2.3 RNA and cDNA sample preparation

Total RNA from the male mouse tissues was isolated using TRIzol reagent (Invitrogen, Waltham, MA, USA) according to the manufacturer’s instructions with minor modifications. To avoid any genomic DNA contamination, DNaseI was used (TaKaRa). Total RNA quality was analyzed using a spectrophotometer at 260/280 nm (Nano Drop ND1000 3.8.1 System, Thermo Fisher Scientific, Waltham, MA, USA). The tested mouse tissues included the whole brain, heart, lungs, liver, small intestine, colon, kidneys, spleen, and testis. Total RNA (1 μg) using oligo-d(T) primers was used to prepare first stranded cDNA according to the manufacturer’s instructions (a High Capacity cDNA Reverse Transcription Kit, Thermo Fisher Scientific, Waltham, MA, USA). Briefly, the cDNA reaction mixture was prepared in advance and synthesized from 1 μg of total RNA. The running conditions were confirmed by conventional PCR before qRT-PCR was conducted. All the experiments were conducted on ice, and the remaining RNA and synthesized cDNA were stored at −80 °C for further experiments as described in a previous report [19].

### 2.4 Quantitative qRT-PCR and primer sequences

qRT-PCR was performed to evaluate the relative mRNA expression levels of LPA receptors (LPA1, LPA2, LPA3, LPA4, LPA5, and LPA6) using the CHROMO4™ RT-PCR system with an SYBR Premix Ex Taq™ II qPCR kit (Takara) as follows: pre-denaturation at 95°C (30 s), and 40 cycles (each) at 95°C (10 s), 60°C (10 s), and 72°C (25 s). Clear melting curves were detected to confirm data integrity by increasing the temperature from 72°C to 97°C. The data were calculated and processed using GeneXpression Macro CHROMO4™ by the supplier after three independent tests. A Ct value beyond 35 was considered negative data in this experiment. To normalize the data, β-actin was used as an internal control and the non-template cDNA-mixture was used as a negative control. The primer sequences used in this experiment were synthesized based on the mouse nucleotide database of the National Center for Biotechnology Information (NCBI) under the following accession numbers: LPA1 (NM_010336, NM_172989, and NM_001290486), LPA2 (NM_020028), LPA3 (NM_022983), LPA4 (NM_175271), LPA5 (NM_001163269 and NM_001163268), and LPA6 (NM_175116). The primer sequences used are listed in Table 1.

**Table 1:** Primer Sequences for LPA1-6 Receptors Used for Quantitative Real-Time Polymerase Chain Reaction (PCR) Analysis

Name	Forward primer (5′→ 3′)	Reverse primer (5′→ 3′)	Size (bp)
LPA1	AGCGCAACGAGAACCCTAAT	TGAATGCTACACGGTCACCC	361
LPA2	GCTGGTTATTGCAGCCATCG	ACACCCACGATGAGTGTGAC	309
LPA3	GGTGTCGAAAACGTTGACCG	GATGCGTACGTATACCGCCA	352
LPA4	TGCTTAGAACCCTCCGCAAG	GAAGGTTTGGGGGTCAGAGG	355
LPA5	GTCCCACTGCACGTACAAGA	TGCGAAGGGTGTTACGGAAA	443
LPA6	GCGCCTGCAGTTTTCTTTCA	TCGGGTACATGGTCCTCACT	379
β-actin	ATGCCATCCTGCGTCTGGACCTG	GCGCTCAGGAGGAGCAATGATCTTG	488

### 2.5 Protein sample preparation

For the overall procedure, the Mem-Per plus kit (Thermo Scientific #89842) was used. About 20–40 mg of tissue was washed with wash buffer, transferred to a 2-mL tissue grinder, and cut into small pieces. After permeabilization buffer was added, the tissues were homogenized (for 6–10 strokes), and the homogenates were transferred to a new tube and incubated for 10 min at 4°C with constant mixing. After centrifugation at 16,000 × *g* for 15 min at 4°C, the supernatant (containing cytosolic proteins) was removed and transferred to a new tube (cytosolic proteins). The resuspended pellet was incubated for 30 min at 4°C with constant mixing. After centrifuging at 16,000 × *g* for 15 min at 4°C, the supernatant (containing the solubilized membrane and membrane-associated proteins) was transferred to new tubes. For all procedures, protease inhibitor cocktail and phosphatase inhibitor cocktail were used to avoid the degradation of the desired proteins. The proteins were either stored in aliquots at −80°C for future use or used immediately.

### 2.6 Western blot

About 30 μg of proteins from the lyzed mice tissues (whole brain, liver, small intestine, and testis) were used. LPA6 (1:5000) was detected via 10% sodium dodecyl sulfate polyacrylamide gel electrophoresis (SDS-PAGE) and blotted onto a 0.45-μm PVDF membrane. The blotted membrane was stripped and re-probed with mouse anti-β-actin monoclonal antibody conjugated with HRP (1:25000). Images were visualized using the Clarity Western ECL Substrate (Bio-Rad, Hercules, CA, USA) on the iBright CL1000 (Thermo Fischer Scientific, MA, United States). Chemi-documentation was conducted for further documentation preservation followed by densitometry analysis of the blots using the program supported by the iBright CL1000.

### 2.7 Statistical analysis

All values are presented as either the mean ± SEM or % of control. A *p* value < 0.05 was considered statistically significant. Significant differences between the means of the control and treatment groups were compared using unpaired Student’s *t*-test or one-way ANOVA.

## 3 Results

### 3.1 Basal gene expression of the six LPA receptor subtypes in the various mouse organs

**Fig. 1:**
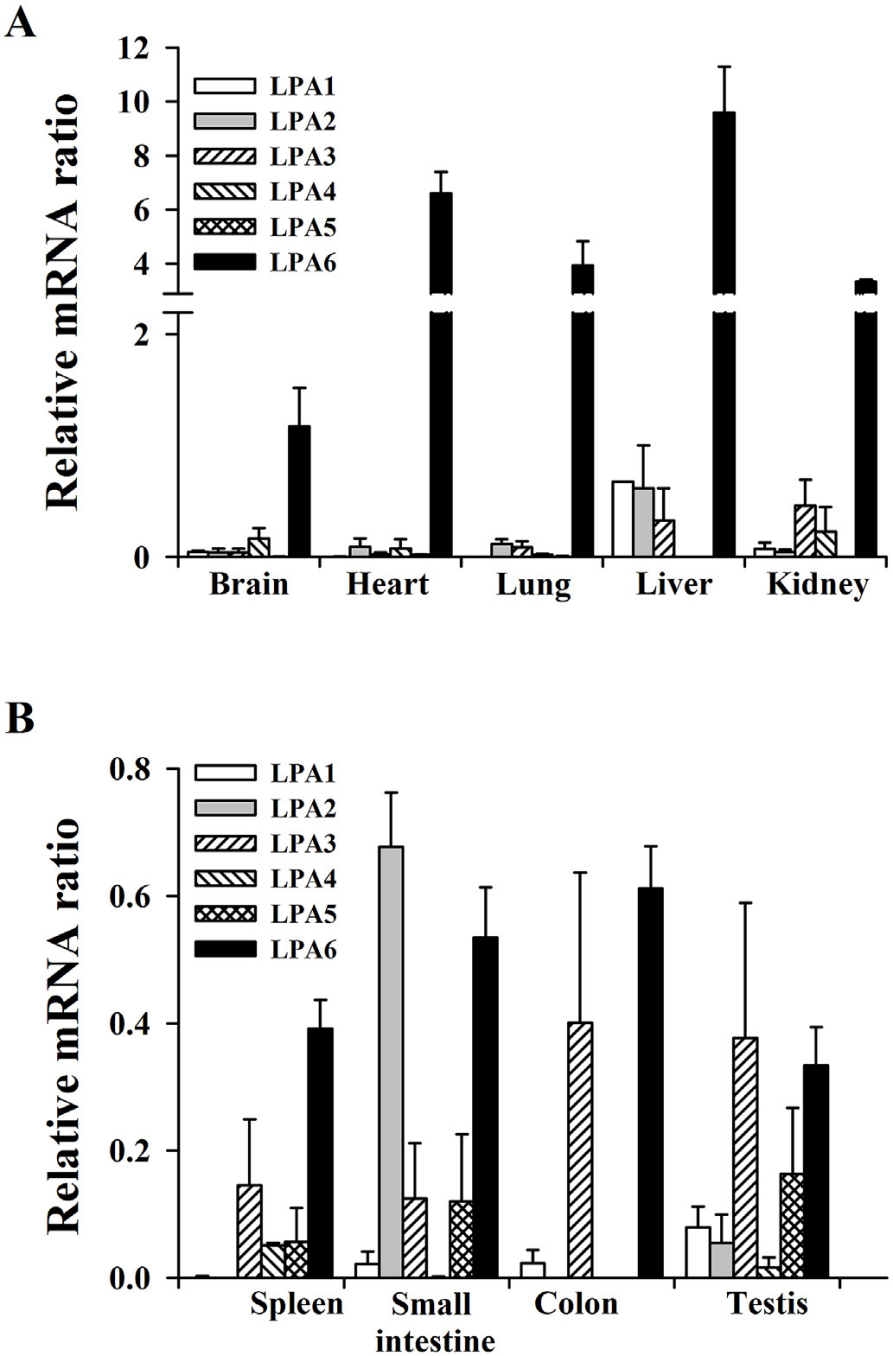
Organ distribution analysis of mouse lysophosphatidic acid (LPA) receptor subtypes (LPA1–LPA6) in mouse organs, A. LPA receptor subtype distributions in the whole brain, heart, lungs, liver, and kidneys. Overall, LPA6 shows the highest expression levels in all tissues. LPA1 expression is very low in all tissues, and LPA2 is expressed mainly in the liver and small intestine. LPA3 is expressed at a low level in general, and LPA4 is detectable in the whole brain, heart, lungs, and kidneys. B. LPA receptor distributions in the spleen, small intestine, colon, and testis. LPA1 expression is very low overall, and LPA2 is expressed mainly in the small intestine. LPA3 is expressed at a low level in general, and LPA4 is detectable in the spleen and testis. In the liver and kidneys, LPA5 is not expressed. The expression patterns from the highest to the lowest are as follows: LPA6; liver > heart > lungs > kidneys > whole brain > colon > small intestine > spleen > testis. LPA2 is specifically highly expressed in the small intestine, whereas the spleen and colon do not show LPA2 expression. Nine mouse organs were used to determine the mRNA expression levels of independent LPA receptors. Total RNA from the tissues was transcribed to first stranded cDNA; this was followed by quantitative real-time PCR analysis using a CHROMO4™ real-time PCR system and a SYBR Premix Ex Taq™ II qPCR kit. The graph is representative of three independent tests (n = 5).

We first examined the normal gene expression patterns of the six LPA receptor subtypes in the major mouse organs, including the whole brain, heart, lungs, kidneys, spleen, small intestine, and colon. We observed that the majority of the organs predominantly expressed the LPA6 receptor subtype, except for the small intestine and testis (Fig. 1). For example, the LPA6 receptor subtype in the liver is expressed more than 8-fold that in the whole brain (Fig. 1). Only the small intestine and testis expressed more LPA2 and LPA3 receptor subtypes than LPA6. The abundance of the LPA6 receptor subtype was as follows: liver > heart > lung = kidney > whole brain > colon = small intestine > spleen = testis (Fig. 1). The colon and testis also expressed the LPA3 as well as LPA6 receptor subtype. Interestingly, the LPA5 receptor subtype was negligibly expressed in all organs examined except in the small intestine, spleen, and testis (Fig. 1). From the normal gene expression study of the six LPA receptor subtypes, we observed two characteristics. First, the LPA6 receptor subtype is abundantly expressed in most of the major organs. Second, the LPA5 receptor subtype is not expressed in most organs except the testis. These results showed that the gene expression patterns of the LPA receptor subtypes in each organ are different from each other. Thus, the six LPA receptor subtypes are differentially expressed in mouse organs.

### 3.2 Long-term effects of GEF on the gene expression of the six LPA receptor subtypes in the various mouse organs

**Fig. 2:**
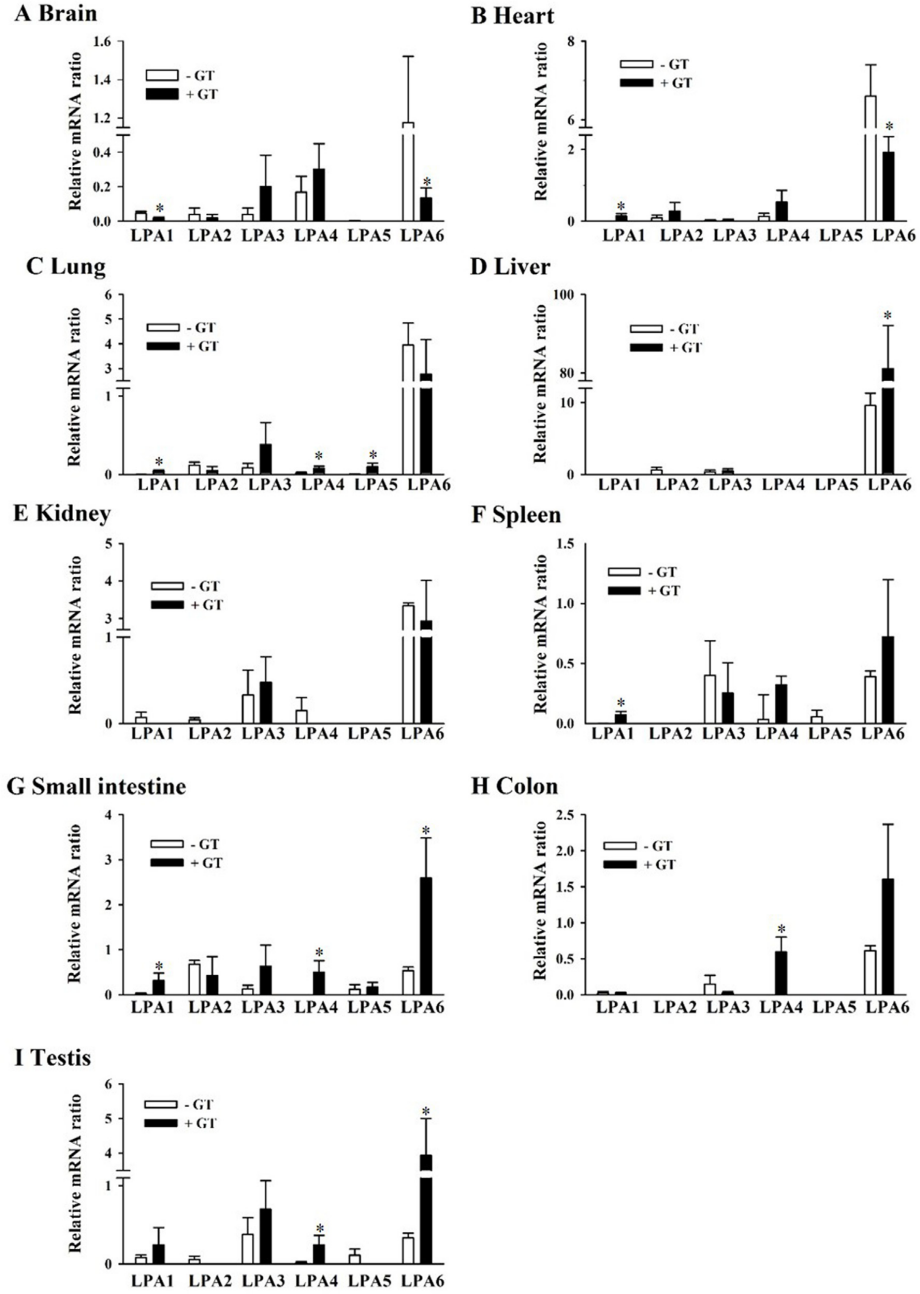
GEF-mediated changes in LPA receptor subtype mRNA expression in nine organs, GEF-mediated changes in LPA receptor subtypes are described in the Results section in detail. Nine mouse organs from both the control and gintonin-treated groups were subjected to qRT-PCR using the CHROMO4™ real-time PCR system and a SYBR Premix Ex Taq™ II qPCR kit as described in Fig. 1.

We examined gene expression changes in the six LPA receptor subtypes after oral administration of GEF for 3 weeks. In the whole brain, the gene expression of the LPA1 and LPA2 receptor subtypes decreased slightly after GEF administration. The LPA3 and LPA4 receptor subtypes increased by 1–2-fold without changes in the LPA5 receptor subtype. However, the LPA6 receptor subtype was significantly decreased by 8-fold in the brain (Fig. 2A; Table 2). In the heart, the gene expression of the LPA1, LPA2, LPA3, and LPA4 receptor subtypes increased after GEF administration by 2–4-fold, whereas that of the LPA6 receptor subtype decreased significantly by 3-fold after GEF administration (Fig. 2B; Table 2). In the lungs, the gene expression of the LPA1, LPA3, LPA4, and LPA5 receptor subtypes increased after GEF administration by 39-, 2-, 4-, and 25-fold, respectively, whereas that of the LPA6 receptor subtype decreased slightly after GEF administration (Fig. 2C; Table 2). Interestingly, long-term administration of gintonin dramatically increased LPA6 receptor subtype expression by nearly 8-fold in the livers (Fig. 2D; Table 2). In the kidneys, LPA3 receptor subtype gene expression increased after GEF administration by 3-fold, whereas LPA4 and LPA6 receptor subtype gene expression decreased slightly after GEF administration. Interestingly, the LPA receptor subtypes in the kidneys were not significantly affected by GEF treatment (Fig. 2E; Table 2). In the spleen, LPA1, LPA4, and LPA6 receptor subtype gene expression increased non-significantly, whereas LPA3 receptor subtype gene expression decreased slightly (Fig. 2F; Table 2). In the small intestine, long-term administration of GEF significantly increased LPA1, LPA4, and LPA6 receptor subtype gene expression. In particular, LPA6 receptor subtype gene expression increased in the small intestine by 4.8-fold (Fig. 2G; Table 2). In the colon, long-term administration of GEF significantly increased LPA4 receptor subtype gene expression (Fig. 2H; Table 2). In the testis, long-term administration of GEF significantly increased LPA4 and LPA6 receptor gene expression. Interestingly, LPA6 receptor subtype gene expression increased about 12-fold after GEF treatment. Thus, these results indicate that long-term administration of GEF affects LPA receptor subtype gene expression in a differential manner depending on the organ type. In Table 2, we have summarized the changes in LPA receptor subtype gene expression before and after GEF administration.

**Table 2:** Expression Values of LPARs After Gintonin Administration

Receptor		Organs									
		Brain		Heart		Lung		Liver		Kidney	
		Mean ± SEM	Fold changes	Mean ± SEM	Fold changes	Mean ± SEM	Fold changes	Mean ± SEM	Fold changes	Mean ± SEM	Fold changes
LPA1	Con	0.045 ± 0.011	2.4 ↓	0.004 ± 0.001	32 ↑	0.001 ± 0.000	39 ↑	ND	ND	0.071 ± 0.060	0 ↓
	GT	0.018 ± 0.006∗		0.143 ± 0.072∗		0.044 ± 0.015∗		ND		0.000 ± 0.000	
LPA2	Con	0.040 ± 0.037	1.8 ↓	0.090 ± 0.076	3 ↑	0.116 ± 0.041	2.3 ↓	0.618 ± 0.382	0 ↓	0.043 ± 0.024	0 ↓
	GT	0.020 ± 0.020		0.278 ± 0.239		0.050 ± 0.050		0.000 ± 0.000		0.000 ± 0.000	
LPA3	Con	0.039 ± 0.037	5 ↑	0.022 ± 0.018	1.4 ↑	0.085 ± 0.056	4.5 ↑	0.329 ± 0.289	1.4 ↑	0.329 ± 0.289	1.4 ↑
	GT	0.200 ± 0.184		0.030 ± 0.030		0.382 ± 0.280		0.478 ± 0.294		0.478 ± 0.294	
LPA4	Con	0.168 ± 0.092	1.8 ↑	0.135 ± 0.082	3.9 ↑	0.019 ± 0.010	4 ↑	ND	ND	0.150 ± 0.150	0 ↓
	GT	0.302 ± 0.147		0.529 ± 0.332		0.077 ± 0.028∗		ND		0.000 ± 0.000	
LPA5	Con	0.002 ± 0.001	0 ↓	0.003 ± 0.002	0 ↓	0.004 ± 0.004	25 ↑	ND	ND	ND	ND
	GT	0.000 ± 0.000		0.000 ± 0.000		0.094 ± 0.049∗		ND		ND	
LPA6	Con	1.176 ± 0.344	8.7 ↓	6.597 ± 0.801	3.4 ↓	3.952 ± 0.882	1.4 ↓	9.589 ± 1.709	8.5 ↑	3.339 ± 0.074	1.1 ↓
	GT	0.135 ± 0.057∗		1.918 ± 0.442∗		2.777 ± 1.397		81.12 ± 11.000∗		2.925 ± 1.091	

Receptor		Organs							
		Spleen		Small Intestine		Colon		Testis	
		Mean ± SEM	Fold changes	Mean ± SEM	Fold changes	Mean ± SEM	Fold changes	Mean ± SEM	Fold changes
LPA1	Con	0.001 ± 0.001	40 ↑	0.022 ± 0.020	14 ↑	0.024 ± 0.021	1.1 ↓	0.080 ± 0.032	3 ↑
	GT	0.073 ± 0.028∗		0.314 ± 0.167∗		0.021 ± 0.010		0.243 ± 0.222	
LPA2	Con	ND	ND	0.677 ± 0.085	1.5 ↓	ND	ND	0.056 ± 0.045	0 ↓
	GT	ND		0.427 ± 0.422		ND		0.000 ± 0.000	
LPA3	Con	0.401 ± 0.236	1.5 ↓	0.125 ± 0.087	5.1 ↑	0.145 ± 0.126	6.7 ↓	0.377 ± 0.212	1.8 ↑
	GT	0.252 ± 0.252		0.635 ± 0.464		0.022 ± 0.017		0.698 ± 0.365	
LPA4	Con	0.035 ± 0.017	9 ↑	0.001 ± 0.001	581 ↑	ND	ND	0.016 ± 0.016	15 ↑
	GT	0.324 ± 0.070		0.499 ± 0.257∗		0.595 ± 0.203∗		0.243 ± 0.122∗	
LPA5	Con	0.056 ± 0.054	0 ↓	0.121 ± 0.105	1.4 ↑	ND	ND	0.109 ± 0.081	0 ↓
	GT	0.000 ± 0.000		0.169 ± 0.103		ND		0.000 ± 0.000	
LPA6	Con	0.392 ± 0.045	1.8 ↑	0.535 ± 0.079	4.8 ↑	0.612 ± 0.067	2.6 ↑	0.334 ± 0.060	11.8 ↑
	GT	0.723 ± 0.476		2.595 ± 0.887∗		1.604 ± 0.765		3.939 ± 1.066∗	

ND: Not detected in two groups.

↓: Tendency to decrease in gintonin-treated group.

↑: Tendency to increase in gintonin-treated group.

∗*p* < 0.05 vs control.

### 3.3 Confirmation of changes in protein levels by western blotting

**Fig. 3:**
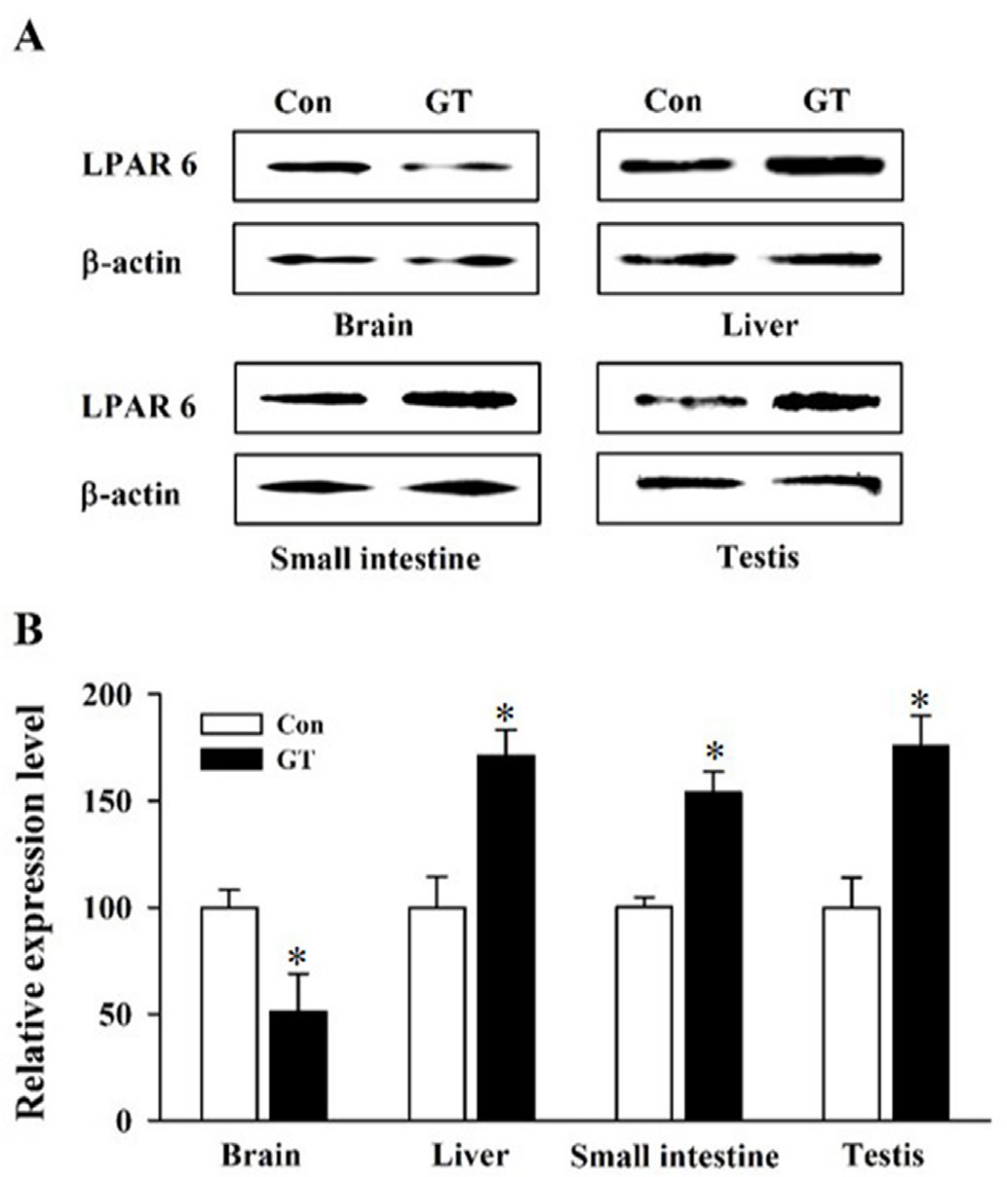
Protein expression of the LPA6 receptor subtype detected by western blot analysis. A. LPA6 receptor protein level was determined using western blotting with representative mouse organs. Either vehicle control or GEF 100 mg/kg (GT) was orally administered to mice, and protein from the whole brain, liver, small intestine, and testis was extracted. A significant increase in the LPA6 protein level was observed in the GEF-treated group (GEF 100 mg/kg) in the liver, small intestine, and testis, whereas it decreased in the whole brain. β-Actin was used as an internal control. The LPA6 protein level was determined using an anti-LPA6 antibody. B. Summary histograms of the LPA6 protein level changes. All values are represented as the mean ± SEM of three independent experiments in triplicate (n = 3). ∗p < 0.05.

In addition, western blot analysis was performed to determine the protein level of the LPA6 receptor subtype because this subtype was recently identified and is predominantly expressed in all organs examined. Long-term GEF administration significantly decreased LPA6 receptor subtype expression at the protein level in the whole brain, whereas long-term GEF administration significantly increased LPA6 receptor subtype protein expression in the liver, small intestine, and testis (Fig. 3A and B). Thus, western blotting results for the LPA6 receptor protein expression analysis were consistent with the qRT-PCR results.

## 4 Discussion

In previous reports, we showed that acute treatment of neuronal and non-neuronal cells with GEF induces transient [Ca^2+^]i through LPA1 receptor activation, which is further coupled to intracellular and intercellular communication for ion channel regulation, neurotransmitter and hormone release, cell survival, cell proliferation, and migration [18]. We also showed that these in vitro gintonin-mediated cellular effects via LPA receptors are linked to beneficial effects in animal models from peripheral corneal wound healing, hair growth promotion, and anti-atopic effects to neurodegenerative diseases such as Alzheimer’s disease (AD), Parkinson’s disease (PD), and Huntington’s disease [9,13,[20], [21], [22], [23], [24]]. Through qRT-PCR and western blot analyses, we further showed that long-term oral administration of GEF increased the gene expression of hippocampal choline acetyltransferase but decreased tryptophan 2,3-dioxygenase in the mouse hippocampus [19]. These results suggest that long-term intake of GEF might help increase brain acetylcholine for cognitive improvement and decrease neurotoxic kynurenic acid formation [25]. However, it is unknown whether long-term administration of GEF affects LPA receptor subtype gene expression in various organs, including the brain. In the present study, we used qRT-PCR and western blot analyses to elucidate the GEF-mediated regulation of LPA receptor subtype gene expression [19]. Here, we examined gene expression changes in nine mouse organs using qRT-PCR and western blot analyses to confirm differential gene expression after the oral administration of GEF.

In this study, we first examined the basal gene expression of the six LPA receptor subtypes (i.e., before GEF administration). We found that most organs showed much higher gene expression of the LPA6 receptor subtype compared to the other LPA receptor subtypes, whereas the gene expression of the LPA5 receptor subtype was much lower in most of the organs except in the testis (Fig. 1; Table 2). Interestingly, the gene expression level of the LPA6 receptor subtype was dominant in the following order: liver > heart > lung = kidney, among the organs. After the LPA6 receptor subtype, the small intestine expressed the LPA2 receptor subtype and the colon and testis expressed the LPA3 receptor subtype, showing that the six LPA receptor subtypes are differentially expressed in each mouse organ.

Next, when we orally administered GEF to the mice for 3 weeks, we noted some significant changes in the gene expression patterns of the LPA receptor subtypes. Interestingly, long-term treatment with GEF decreased LPA6 receptor subtype gene expression in the whole brain, heart, lung, and kidney but increased it in the liver, small intestine, colon, and testis. Thus, long-term GEF treatment differentially regulates the LPA6 receptor subtype, depending on the organ type. In addition to the LPA6 receptor subtype, the gene expression levels of the LPA3 receptor subtype increased after GEF administration, except in the colon and intestine (Table 2). The gene expression level of the LPA4 receptor subtype increased after GEF administration, except in the liver, kidney, and colon (Table 2). The gene expression levels of the other LPA receptor subtypes (such as the LPA1 and LPA2 receptor subtypes) were variable after GEF administration and dependent on the organ type (Table 2). In western blotting experiments, to confirm the changes in the protein expression of the LPA receptor subtypes after GEF administration, we chose the LPA6 receptor subtype because this receptor subtype was most abundantly expressed in all the organs examined. We found that, after GEF treatment, the protein level of the LPA6 receptor decreased in the brain, liver, and small intestine but increased in the testis, which is consistent with the qRT-PCR results (Fig. 3). Although long-term GEF administration induced changes in the gene expression levels of the six LPA receptor subtypes, we did not elucidate the manner in which the GEF-mediated gene expression changes in the individual LPA receptor subtypes are linked to the physiological and/or pharmacological effects of GEF. However, in previous preclinical studies, we showed that long-term GEF administration had beneficial effects on nervous and non-nervous systems [9,[19], [20], [21], [22], [23], [24],^26^]. Thus, further studies are required to elucidate the physiological and pharmacological relevance of the GEF-mediated regulation of LPA receptor subtype expression in each organ.

Six LPA receptor subtypes have been identified. They are divided into two classes of genes. One is the EDG family [LPA1 (EDG2), LPA2 (EDG4), and LPA3 (EDG7)] and the other is the non-EDG family [LPA4 (GPR23/P2Y9), LPA5 (GPR92), and LPA6 (P2Y5)]; this classification is based on the large differences in their chemical structures, though they all recognize and bind to LPAs [27]. The LPA1 receptor subtype was first identified from a neuronal cell line, and the other LPA receptor subtypes were found in nervous and non-nervous systems [28]. These activated LPA receptors are coupled to several types of GTP-binding proteins to initiate diverse downstream signaling pathways such as the activation of the phospholipase C, Rho, Akt, and phosphatidylinositol 3-kinase pathways or the regulation of adenylyl cyclase [29]. This in turn mediates in vivo biological effects, as described in the Introduction.

Interestingly, the LPA6 receptor subtype is the most recently identified member of the LPA receptor family in Xenopus, and it was reported that the LPA6 receptor is expressed in developing telencephalon [30,31]. A previous report showed that the LPA6 receptor subtype gene is expressed at a greater extent than any other LPA receptor subtype in the mouse brain, and its expression level was as high as that of the LPA1 receptor subtype from mouse brain development to adult brain stages [32]. The authors of the previous study also observed LPA6 receptor subtype gene expression in primary neurons, immature and mature astrocytes, and microglia [32,33]. Another study also reported LPA6 receptor subtype mRNA expression in the neocortex, hippocampus, cerebellum, and olfactory bulbs [33]. In the present study, we found that the LPA6 receptor subtype was expressed predominantly in all organs compared to the other LPA receptor subtypes, even in adult mice (Fig. 1). Thus, we provide evidence that the LPA6 receptor subtype is also predominantly expressed in all other organs examined as well as in the brain in adult mice.

Interestingly, although most mouse organs abundantly express the LPA6 receptor subtype compared to the other subtypes, its physiological functions have not yet been majorly elucidated. The LPA and LPA6 subtype might play an important role in human hair growth because congenital mutations in the LPA6 receptor cause abnormal hair growth in humans, such as hypotrichosis simplex [26,34,35]. Recently, rat brain capillary endothelial cells were reported to predominantly express the LPA6 receptor gene. Treatment of brain capillary endothelial cells with LPA increases blood–brain barrier permeability via the LPA6-Gα_12/13_ and Rho kinase pathways. The knockdown of the LPA6 receptor subtype caused the blockade of LPA-mediated increases in blood–brain barrier permeability [36]. These reports show that the LPA6 receptor subtype might play important roles in the nervous and non-nervous systems. In previous reports, although we showed that long-term GEF treatment stimulated mouse hair growth and acute gintonin treatment enhanced the blood–brain barrier permeability of low- and high-molecular-weight drugs and hormones, we could not pharmacologically demonstrate the gintonin- or GEF-mediated effects achieved via LPA6 receptor regulation because there is no selective LPA6 receptor antagonist available similar to that available for the other LPA receptor subtypes [9,13,26]. In the future, further studies are required to elucidate the involvement of the LPA6 receptor subtype in GEF-mediated biological effects in various body organs.

In summary, among the six LPA receptor subtypes, the LPA6 receptor subtype showed a predominant expression in all the organs examined. The oral administration of GEF affected the gene expression of all six LPA receptor subtypes in a differential manner depending on the organ type. Although the functions of the LPA receptor subtypes are currently not well characterized in organs except in the brain, the present study indicates that genes related to the six LPA receptor subtypes can be regulated by GEF administration. Finally, long-term GEF administration can affect the gene expression of the six LPA receptor subtypes in adult mouse organs, though further studies might be required to confirm how the GEF-mediated regulation of the six LPA receptor subtypes is linked to the biological effects of GEF in nervous and non-nervous systems.

## Declaration of competing interest

All authors have no conflicts of interest to declare.
